# Highly Efficient Prion Transmission by Blood Transfusion

**DOI:** 10.1371/journal.ppat.1002782

**Published:** 2012-06-21

**Authors:** Olivier Andréoletti, Claire Litaise, Hugh Simmons, Fabien Corbière, Séverine Lugan, Pierrette Costes, François Schelcher, Didier Vilette, Jacques Grassi, Caroline Lacroux

**Affiliations:** 1 UMR INRA ENVT 1225, Interactions Hôtes Agents Pathogènes, Ecole Nationale Vétérinaire de Toulouse, Toulouse, France; 2 VLA Weybridge, ASU, New Haw, Addlestone, Surrey, United Kingdom; 3 CEA, Service de Pharmacologie et d'Immunoanalyse, IBiTec-S, DSV, CEA/Saclay, Gif sur Yvette cedex, France; Creighton University, United States of America

## Abstract

It is now clearly established that the transfusion of blood from variant CJD (v-CJD) infected individuals can transmit the disease. Since the number of asymptomatic infected donors remains unresolved, inter-individual v-CJD transmission through blood and blood derived products is a major public health concern. Current risk assessments for transmission of v-CJD by blood and blood derived products by transfusion rely on infectious titers measured in rodent models of Transmissible Spongiform Encephalopathies (TSE) using intra-cerebral (IC) inoculation of blood components. To address the biological relevance of this approach, we compared the efficiency of TSE transmission by blood and blood components when administrated either through transfusion in sheep or by intra-cerebral inoculation (IC) in transgenic mice (tg338) over-expressing ovine PrP. Transfusion of 200 µL of blood from asymptomatic infected donor sheep transmitted prion disease with 100% efficiency thereby displaying greater virulence than the transfusion of 200 mL of normal blood spiked with brain homogenate material containing 10^3^ID_50_ as measured by intracerebral inoculation of tg338 mice (ID_50_ IC in tg338). This was consistent with a whole blood titer greater than 10^3.6^ ID_50_ IC in tg338 per mL. However, when the same blood samples were assayed by IC inoculation into tg338 the infectious titers were less than 32 ID per mL. Whereas the transfusion of crude plasma to sheep transmitted the disease with limited efficacy, White Blood Cells (WBC) displayed a similar ability to whole blood to infect recipients. Strikingly, fixation of WBC with paraformaldehyde did not affect the infectivity titer as measured in tg338 but dramatically impaired disease transmission by transfusion in sheep. These results demonstrate that TSE transmission by blood transfusion can be highly efficient and that this efficiency is more dependent on the viability of transfused cells than the level of infectivity measured by IC inoculation.

## Author Summary

In the UK, several v-CJD cases have been identified in patients that received blood or blood-derived products prepared from incubating asymptomatic donors. Since there is no screening test to identify infected donors, procedural risk reduction measures remain the only protection against v-CJD transfusion risk. These measures rely, in part, on the assumptions that (i) the level of infectivity in blood is low and (ii) the risk of blood borne transmission is directly correlated with the infectious titer of blood and blood products. Using a transmissible spongiform encephalopathy (TSE) animal model, we have provided evidence that despite a very low infectious titer in blood as measured by inoculation into brain, the transfusion of 0.2 mL of blood from asymptomatic infected donors is sufficient to transmit the disease with a 100% efficacy. We further demonstrated that this high efficiency of disease transmission is crucially dependant on the viability of the transfused white blood cells rather than on their infectious titer. These findings provide new insights into the pathogenesis of TSE diseases and require revision of some of the key assumptions of the v-CJD blood borne risk assessments.

## Introduction

In 1996, a new form of CJD, named variant CJD (v-CJD) was identified in humans [1], [2]. The presence of the v-CJD agent in lymphoid tissues of patients incubating the disease raised the possibility of blood borne v-CJD transmission. Since then, four v-CJD cases were reported in patients transfused with labile blood products from asymptomatic donors who later developed v-CJD [3].

Current measures for prevention of v-CJD transmission by blood products rely on risk assessments based on infectious titers reported for the blood of rodent models of TSE [4]. These infectious titers were established by inoculating blood components by the intracerebral (IC) route in homologous species. All these experiments concur in indicating that infectivity in the blood of symptomatic rodents varies between 1 and 10 ID_50_ per mL and that both plasma and leukocytes were infectious [5], [6], [7], [8], [9], [10].

However, the IC inoculation route is unlikely to reflect the specifics of the transfusion exposure route; *i.e.* the administration of large numbers of living cells and a large volume intravenously to the recipient.

In early 2000, transmission of both experimental BSE and natural scrapie were reported to occur following transfusion of whole blood collected in asymptomatic incubating sheep [11], [12]. The greater similarity in size between humans and sheep allows the transfusion of blood volumes in sheep that are closer to those used in human medicine. Moreover, the pathogenesis of variant CJD in humans is very similar to that in classical scrapie or BSE in sheep. As a consequence sheep TSE models are accepted as relevant for assessing v-CJD transmission risk through the transfusion route [13], [14].

In this study, using a TSE sheep infection model, we investigated the ability of blood and blood components to transmit the disease when administered by the transfusion route and measured their relative infectious titer by IC inoculation in transgenic mice (tg338) over-expressing ovine PrP. Our findings show that the disease is transmitted with high efficiency by transfusion of infected blood and blood components and requires revision of some of the key assumptions used for assessing the risk of blood borne transmission of v-CJD.

## Materials and Methods

### Ethic statement

All animal experiments have been performed in compliance with our institutional and French national guidelines, in accordance with the European Community Council Directive 86/609/EEC. The experimental protocol was approved by the INRA Toulouse/ENVT ethics committee.

### Infection of sheep with classical scrapie strain PG127 by oral inoculation

VRQ/VRQ sheep were produced in a biosecure unit from a flock that was sourced originally from New Zealand by Veterinary Laboratory Agency in the United Kingdom (UK). This TSE free flock is a unique source of VRQ/VRQ animals that are considered free of classical scrapie [15]. The scrapie inoculum was prepared from symptomatic VRQ/VRQ sheep experimentally infected with PG127 scrapie isolate. This stock was previously titrated by end-point dilution in tg338 mice inoculated by the intracerebral (IC) route [16]. TSE-free Cheviot sheep (6–10 months old) were orally challenged with a 2 g equivalent of brain (administered as 1% brain homogenate in glucose). Animals were then observed until the occurrence of clinical signs (around 200 days post inoculation) and euthanized when exhibiting locomotor signs of the disease that impaired feeding. In this model clinical phase (first clinical signs occurrence to recumbency) is short (one to two weeks). After culling, each sheep was necropsied and lymphoid tissues (spleen, third eye-lid, mesenteric lymph node, prescapular lymph node, tonsil) were sampled in addition to the CNS for PrP^Sc^ detection. The PrP genotype was obtained for every animal by sequencing Exon 3 of the *Prnp* gene as previously described [17].

### Blood collection and separation

Blood was collected in vigil animals from jugular vein into Macopharma MSE3500 collection bags. To separate plasma, buffy coat and red cells, blood units were centrifuged at 3640 rpm for 12 minutes (including acceleration and deceleration) at 20°C in a Jouan LR5-22 centrifuge. For each sample, packed cell volume and cell numeration were measured using IdeXX Vet-station automat and Sysmex XT2000i. White blood cells (WBC) were obtained from buffy coat fraction, by osmotic lysis of erythrocytes with ACK solution (NH4CL 0,15 M, KHCO3 1 mM, Na2EDTA 0,1 mM, pH 7.4). Briefly, buffy coat (1 volume) was gently mixed (by rolling tubes) with ACK (1 volume) and incubated for 5 min RT. WBC were then washed 3 times with 50 mL PBS to remove erythrocytes debris and plasma.

WBC fixation was performed using paraformaldehyde (PFA)/PBS solution. WBC cells were re-suspended in a 50 mL conic tube using 10 mL of PBS and 10 mL of a PBS/PFA 4% solution was added (final concentration 2%). Tubes were then rolled on bench during 20 seconds and incubated for 10 min on ice. Cells were then washed four times in PBS (50 mL) and their morphology (lack of clumps and cellular integrity) was checked by direct microscopic examination and flow cytometry.

### Transfusion and intravenous administration

Recipient sheep were anesthetized by IV administration of Ketamine/Diazepam (2 mg and 0.1 mg per kg respectively) and a catheter (16G) was installed in the jugular vein. This catheter was used for all transfusions and intravenous administrations of blood and blood products. Whole blood (200 mL), plasma (100–150 mL) or WBC (re-suspended in 100 mL 5% glucose) were transfused/intravenously administered by gravity in individual recipients over a 30–40 min period.

In the whole blood spiking experiment, the titrated PG127 brain homogenate was diluted in isotonic (5%) glucose solution to obtain the desired number of Infectious Dose 50 as measured by IC inoculation in tg338 mice (ID_50_ IC in tg338) in a 20 mL volume. Each inoculum was then mixed with 200 mL of freshly collected whole blood by 30 minutes of continuous rocking before transfusion back into the same donor sheep. In the whole blood titration by transfusion route, the required whole blood volumes were sampled from the collection bag using a sterile syringe and either transferred into a new pouch or gently re-suspended in a sterile 5% glucose solution (QSP 200 mL), before administration (gravity) to recipients. In the WBC experiment, WBC (fresh or fixed) were gently re-suspended in a 200 mL sterile pouch of isotonic (5%) glucose before infusion (gravity) over a 20–30 min period. All transfusions were performed within the 6 hours following blood collection.

### Sheep monitoring and necropsy

Sheep were housed in a category 2 animal facility. Donor and recipient sheep were kept separated. Contact controls (n = 5) were housed in the same pen as transfusion recipients throughout the experiment. None displayed any TSE symptoms. Orally inoculated sheep (donors) and transfused sheep (recipients) were monitored daily. When displaying symptoms that could impair their ability to feed, the animals were euthanized by an intravenous injection of T61 (Intervet). Transfused animals that had not displayed clinical signs after 400–450 days were also euthanized. Upon completion of the experiment, the contact controls were also euthanized. All animals were systematically necropsied and a panel of tissues (brain, spleen, mesenteric lymph node, tonsil, third eyelid, prescapular lymph node, ileum was collected. Each sample was divided in two parts, the first one was formalin fixed for abnormal PrP (PrP^Sc^) immunohistochemistry investigation while the other was snap frozen at −80°C for WB PrP^Sc^ detection.

### Bioassay in tg338 mice

Bioassay experiments were carried out in transgenic mice (tg*338*) over-expressing the VRQ allele of the ovine prion protein (PrP) [18]. At least six mice were inoculated intracerebrally with each sample (20 µL). Mice were then monitored for the appearance of TSE clinical signs. Mice that did not succumb to TSE were euthanized at 200 days post inoculation (dpi). The last positive transmission was observed 150 dpi during an end-point dilution titration of a PG127 brain stock [16]. CNS and spleen samples collected from each individual animal were tested by Western blot (WB).

Whole blood, plasma and red blood cell concentrate were high speed homogenized for 90 seconds (Precess 48- Bertin, France) and inoculated neat (20 µl IC) in tg338 (n = 18). No acute toxicity was observed at inoculation during this experiment. The infectious titer per mL of blood (number of ID/mL) was estimated by the limiting dilution titration (application of Poisson model) described by *Brown et al* to estimate the infectious titer in TSE affected rodents' blood and classically used in this type of study [5], [7]. Percentage volume of packed blood cells and plasma were used to calculate the infectious titer per mL of blood.

WBC corresponding to known quantity of whole blood was high speed homogenized for 90 seconds (Precess 48- Bertin, France) in 5% glucose solution. The resulting suspension was then titrated by end point dilution by intracerebral (IC) inoculation of 1/10 successive dilutions into groups of 6 tg*338* mice (20 µL/mouse). The infectious titer (Infectious Dose 50, most likely value and 95% confidence intervals) were determined by the Spearman-Kärber method [19].

According to Fischer [20] and as previously used for Prion infectivity ID/ID50 comparison [21] one ID_50_ per mL was considered in average equivalent to 0.693 ID.

### PrP^Sc^ Western-blot (WB) and immunohistochemistry

A Western blot kit (TeSeE Western Blot, Bio-Rad) was used following the manufacturer's recommendations. For each sample, 250 µL of 10% tissue homogenate was subjected to PrP^Sc^ extraction. Immunodetection was performed using Sha31 monoclonal antibody conjugated to horseradish peroxidase (0,06 µg per mL) which recognizes amino acids 145–152 (YEDRYYRE) of the sheep PrP sequence. Peroxidase activity was detected using ECL substrate (Pierce). Immunohistochemistry was performed as previously described [22], using 8G8 antibody which specifically recognises amino acids 95–108 (SQWNKP) of the sheep PrP protein.

## Results

The first experiment was to determine the minimum infectious dose (ID_50_ as measured by end point dilution titration in tg338 mice by the IC route) able to transmit TSE infection in sheep by transfusion. This was done by transfusing groups of two TSE free VRQ/VRQ recipient sheep with whole blood that had been spiked with successive 1/10 dilutions of a brain homogenate prepared from scrapie affected sheep (PG127 isolate). This brain homogenate had been previously titrated by end point dilution in tg338 mice, and had a titer of 10^6.6^ ID_50_ IC/g [16]. The lowest dose resulting in transmission was observed in sheep (1 infection out of 2 challenged individuals) that were transfused with blood containing 10^3^ ID_50_ IC in tg338 mice (Table 1). No clinical signs or abnormal PrP deposition were observed in animals that were challenged with lower doses and observed for at least 750 days post challenge.

**Table 1:** Transfusion of VRQ/VRQ sheep with blood spiked with decreasing amounts of PG127 infected sheep brain homogenate.

	10^6^ ID_50_ IC in tg338		10^5^ ID_50_ IC in tg338		10^4^ ID_50_ IC in tg338		10^3^ ID_50_ IC in tg338		10^2^ ID_50_ IC in tg338		10 ID_50_ IC in tg338	
	dpi	PrP^Sc^	dpi	PrP^Sc^	dpi	PrP^Sc^	dpi	PrP^Sc^	dpi	PrP^Sc^	dpi	PrP^Sc^
**Recipient 1**	171	+	186	+	202	+	224	+	>750	−	>750	−
**Recipient 2**	181	+	196	+	220	+	>750	−	>750	−	>750	−

200 mL of whole blood was collected from each of 12 TSE Free VRQ/VRQ cheviot sheep. Whole blood pouches were spiked with decreasing amounts of sheep scrapie strain PG127 brain homogenate containing 10^6.6^ ID_50_/g as measured by IC inoculation in tg*338* mice. Each spiked blood was then transfused back into the sheep of origin. Two sheep were challenged at each dose (indicated as number of ID_50_ IC in tg338). Sheep were observed until they developed clinical signs or reached 750 days post inoculation when they were euthanized. All recipients were tested for presence of abnormal PrP (PrP^Sc^) deposition in brain and various lymphoid tissues by immunohistochemistry. Results confirmed the clinical diagnosis. Incubation periods in recipients are presented as days post inoculation (dpi).

In parallel, seven VRQ/VRQ sheep were orally challenged with 10^6.7^ ID_50_ IC in tg338 of the same PG127 brain homogenate stock. Whole blood samples were collected in four of these animals (D1–D4) at a late stage of the incubation period (two to five weeks before clinical onset). 200 mL, 20 mL, 2 mL and 0.2 mL of each donor's fresh whole blood were transfused into TSE free VRQ/VRQ recipients (Table 2). All animals that received 200 mL, 20 mL or 2 mL of blood developed clinical TSE. Three out of the four recipients that received the 0.2 mL dose also developed clinical scrapie. The fourth recipient was apparently healthy when euthanized after 450 days of incubation but had abnormal PrP depositions in both lymphoid tissues and the central nervous system.

**Table 2:** Titration of whole blood from PG127 scrapie infected sheep by transfusion into VRQ/VRQ TSE-free recipient sheep.

	200 mL		20 mL		2 mL		0.2 mL	
	dpi	PrP^Sc^	dpi	PrP^Sc^	dpi	PrP^Sc^	dpi	PrP^Sc^
**D1**	188	+	197	+	207	+	210	+
**D2**	176	+	184	+	186	+	203	+
**D3**	173	+	203	+	214	+	>450	+
**D4**	169	+	178	+	193	+	214	+

Four scrapie susceptible VRQ/VRQ sheep between 6 and 10 months of age (identified as D1, D2, D3 and D4) were orally challenged with 2 grams of sheep-scrapie strain PG127 infected brain homogenate containing 10^6.6^ ID_50_/g as measured by IC inoculation of tg338 mice. These animals were euthanized at 227 days, 256 days, 221 days and 226 days post inoculation (dpi) respectively with symptomatic scrapie. Scrapie infection was confirmed by histopathological detection of vacuolar changes in the central nervous system and by immunohistochemical detection of abnormal PrP deposits in the central nervous system and lymphoid tissues. Whole blood had been collected from each donor at 210 dpi. Donors developed clinical scrapie two to five weeks following blood collection. 200 mL, 20 mL, 2 mL and 0.2 mL were intravenously administrated (jugular vein) to TSE Free Cheviot VRQ/VRQ recipients, within 6 hours following collection. Recipients were observed until occurrence of clinical signs. At 450 days post inoculation the single recipient that was still alive was euthanized. This animal was clinically normal and healthy. All transfusion recipients were tested for presence of abnormal PrP deposition in brain and various lymphoid tissues by immunohistochemistry and western blot. All recipients, including the asymptomatic survivor to 450 days displayed evidence of infection. Incubation periods in recipients are presented as days post inoculation (dpi).

These results, when considered together, indicate that the transfusion of 0.2 mL of whole blood transmitted scrapie more efficiently than the IV administration of 10^3^ ID_50_ IC in tg338 (brain homogenate material). It therefore suggested that in scrapie infected sheep (close to symptomatic phase) the infectious load per mL of blood should be higher than 10^3.6^ ID_50_ IC in tg338.

To confirm this estimate, whole blood from three donors (D1 to D3) and one TSE free control (C1) were each inoculated intracerebrally into eighteen tg338 mice (20 µL per mouse). An incomplete attack rate was observed in the inoculated mice (Table 3) and infectious titers were estimated by a statistical method previously used to calculate the concentration of infectivity in blood of TSE infected rodents models [5], [23]. Whole blood titers ranged between 3 and 16 ID IC in tg338 mice per mL (Table 3).

**Table 3:** Intracerebral inoculation of tg338 mice with VRQ/VRQ sheep whole blood, plasma and red blood cell concentrate.

Donor	Specimen	Total inoculated volume (equivalent whole blood)	Number of inoculated mice	Number of positive mice	ID per mL of whole blood
**D1**	Whole blood	360 µL	18	5/18	16.3 (5.8–31.6)
	Plasma	529 µL	18	1/18	1.9 (0.1–8.6)
	RBC	1125 µL	18	0/18	0 (0–2.7)
**D2**	Whole blood	360 µL	18	2/18	5.9 (1–18.2)
	Plasma	474 µL	18	0/18	0 (0–6.3)
	RBC	1500 µL	18	0/18	0 (0–2)
**D3**	Whole blood	360 µL	18	1/18	2.9 (0.2–12.6)
	Plasma	507 µL	18	0/18	0 (0–6)
	RBC	1241 µL	18	0/18	0 (0–2.5)
**C1**	Whole blood	360 µL	18	0/18	-

Blood was collected from three TSE free VRQ/VRQ donor sheep that had been orally challenged with PG127 scrapie (D1, D2, D3) and one TSE free VRQ/VRQ control sheep (C1). The date of collection from the infected animals was 210 days post inoculation. Donors developed clinical scrapie two to five weeks following blood collection. They were euthanized at 227 days, 256 days, 221 days respectively. Whole blood, plasma, red blood cell concentrate (RBC) were each inoculated intracerebrally into 18 tg338 mice (20 µL per mouse). For each component, the volume of whole blood corresponding to the volume inoculated is given. Mice were euthanized when they showed clinical signs of infection or after 250 dpi. Mice were considered infected when abnormal PrP depositions were detected in brain. Infectious titers were estimated using limiting dilution titration method (application of Poisson model) described by Brown et al [23]. Infectious titers are given as the most likely infectious titer and, in parentheses, the values of the lower and upper limits of the 95% confidence interval.

Plasma, red blood cells and WBC were prepared from the same blood samples. Plasma and RBC were inoculated undiluted intracerebrally into eighteen tg338 mice. Only a single infection was observed (D1 plasma). The infectivity titers in plasma and red blood cells expressed as the equivalent volume of whole blood were computed to have a 0.95 probability of contributing less than 8.6 and 2.7 ID IC in tg338 per mL of blood respectively.

The infectivity in WBC was measured by end point dilution titration by IC inoculation of ten folds serial dilutions in tg338 mice (Table 4). Titers calculated by the Spearman-Karber method and expressed as the equivalent volume of whole blood ranged between 7 and 24 ID_50_ IC in tg338 per mL of blood, *i.e* between 4.5 and 13.4 ID per mL (one ID_50_/mL being in average equivalent to 0.693 ID/mL).

**Table 4:** Tg338 mice intracerebral inoculation with VRQ/VRQ sheep white blood cells homogenates.

Donor	Specimen	Total inoculated volume (equivalent whole blood)	Number of positive mice	ID_50_ per mL of whole blood
**D1**	WBC neat	9.8 mL	6/6	
	WBC 1/10	980 µL	4/6	19.4 (5.5–67.8)
	WBC 1/100	98 µL	2/6	
	WBC 1/1000	9.8 µL	0/6	
**D2**	WBC neat	9.6 mL	6/6	
	WBC 1/10	960 µL	4/6	13.5 (4.3–41.6)
	WBC 1/100	96 µL	1/6	
	WBC 1/1000	9.6 µL	0/6	
**D3**	WBC neat	9,2 mL	6/6	
	WBC 1/10	920 µL	3/6	6.5 (2.5–16.7)
	WBC 1/100	92 µL	0/6	
**C1**	WBC neat	9.7 mL	0/6	-

Blood was collected from three TSE free VRQ/VRQ donor sheep that had been orally challenged with PG127 scrapie (D1, D2, D3) and one TSE free VRQ/VRQ control sheep (C1). The date of collection from the infected animals was 210 days post inoculation. Donor sheep developed clinical signs within two to five weeks following blood collection. They were euthanized at 227 days, 256 days, 221 days respectively White blood cells (WBC) were prepared from whole blood and homogenised in 5% glucose solution. Successive 1/10 dilutions of WBC homogenates were inoculated intra-cerebrally to tg*338* mice (n = 6). The equivalent volume of whole blood inoculated in mice is indicated. Mice were euthanized when they showed clinical signs of infection or after 250 dpi. Mice were considered infected when abnormal PrP depositions were detected in brain. Infectious titer was estimated by the Spearman-Karber method [19]. Infectious titer is expressed as number of ID_50_ per mL of whole blood. For each samples, the most likely value and (in parentheses) the lower and upper value of the 95% confidence interval are reported.

The infectious titers of whole blood in sheep incubating scrapie were comparable with those previously reported in hamster and mouse models [6], [7], [8], [9], [10]. However, these titers were at least 100 fold lower than those initially suggested by the transfusion experiments.

In an attempt to resolve this discrepancy we investigated the relative abilities of whole blood, plasma and white blood cells to transmit the disease by the transfusion route in sheep. Over 700 mL of blood was collected, few days (D4) to three weeks (D7) before clinical onset, from each of four orally challenged VRQ/VRQ sheep (D4 to D7) (Tables 5, 6). 500 mL of each blood was used to prepare plasma and WBC. The WBCs were divided into two equal parts; the first half was untreated while the other half was fixed with PBS-paraformaldehyde (PFA). Fresh whole blood (200 mL), fresh WBC and PFA fixed WBC (each equivalent to 200 mL of starting blood) and plasma prepared from 200 mL and 20 mL of blood were administrated intravenously to TSE free VRQ/VRQ recipient sheep (Table 5).

**Table 5:** Intravenous administration in sheep and intracerebral challenge in tg338 mice of blood derived products prepared from PG127 scrapie infected sheep.

	IV administration in sheep									
	Whole blood		Plasma (200 mL)		Plasma (20 mL)		Fresh WBC		WBC fixed in 2% PFA	
	dpi	PrP^Sc^	dpi	PrP^Sc^	dpi	PrP^Sc^	dpi	PrP^Sc^	dpi	PrP^Sc^
**D4**	181	+	>450	−	>380	−	184	+	>450	−
**D5**	177	+	235	+	NA		179	+	251	+
**D6**	167	+	259	+	>380	−	181	+	>450	−
**D7**	168	+	199	+	>380	−	169	+	215	+

Four scrapie-susceptible VRQ/VRQ sheep (identified as D4, D5, D6 and D7) were orally challenged with 1 g of sheep scrapie strain PG127 infected brain homogenate containing 10^6.7^ IC ID_50_/g as measured by IC inoculation of tg338 mice Scrapie incubation periods were 226 dpi, 238 dpi, 228 dpi and 242 dpi, respectively. Blood was collected at 217 dpi, *i.e* few days (D4) to three weeks (D7) before clinical onset. Plasma and white blood cells (WBC) were prepared from 500 mL of whole blood. Half of the WBC preparation from each animal was fixed with paraformaldehyde (PFA 2% final concentration). Whole Blood, plasma and both fresh and fixed WBCs (re-suspended in 5% glucose), each corresponding to 200 mL of whole blood, were administered intravenously to VRQ/VRQ TSE free recipients. In addition, Plasma volume equivalent to 20 mL of whole blood was also intravenously administered to sheep. Recipients were euthanized when symptomatic with scrapie. 450 dpi (or 380 dpi for the 20 mL plasma) recipients that were still alive and apparently healthy were euthanized. Incubation periods in recipients are presented in days post inoculation (dpi). All recipient sheep were tested for the presence of abnormal PrP deposition in brain and various lymphoid tissues by immunohistochemistry.NA: not assessed.

**Table 6:** End-point titration in tg338 mice of a 10% brain homogenate and white blood cells samples, collected in VRQ/VRQ sheep orally inoculated with PG127 scrapie.

	Brain*		D4 WBC				D5 WBC				D6 WBC				D7 WBC			
			Fresh		PFA fixed		Fresh		PFA fixed		Fresh		PFA fixed		Fresh		PFA fixed	
Dilution	Pos mice	dpi	Pos mice	dpi	Pos mice	dpi	Pos mice	dpi	Pos mice	dpi	Pos mice	dpi	Pos mice	dpi	Pos mice	dpi	Pos mice	dpi
**neat**	6/6	64±4	6/6	90±1	6/6	92±3	6/6	100±11	6/6	106±6	6/6	97±4	6/6	95±3	6/6	87±3	6/6	85±2
**10^−1^**	6/6	76±3	5/6	107±11	5/6	106±8	ND		ND		ND		ND		4/6	108±13	5/6	117±19
**10^−2^**	6/6	87±2	2/6	111,115^†^	2/6	108,110^†^									1/6	111^†^	0/6	>200
**10^−3^**	6/6	97±5	0/6	>200	0/6	>200								-	0/6	>200	0/6	>200
**10^−4^**	3/6	110±4																
**10^−5^**	2/6	117–121^†^																
**10^−6^**	0/6	>200																
**10^−7^**	0/6	>200																
**Infectious titer**	10^7^ (10^6.4^–10^7.6^) ID_50_/g		18.6 (6–57.5) ID_50_/mL		18.6 (6–57.5) ID_50_/mL		NA		NA		NA		NA		8.6 (2.8–26.7) ID_50_/mL		8.6 (4.3–17.4) ID_50_/mL	

10% weight/volume homogenate (*) was prepared using posterior brainstem from VRQ/VRQ sheep inoculated with PG127 scrapie isolate and at the terminal stage of disease. Groups of 6 mice that over-express the VRQ ovine PrP (tg*338*) were intracerebrally (20 µL) inoculated with successive 1/10 dilutions of this homogenate. These data were already used in a previous publication [16]. In parallel, four susceptible VRQ/VRQ sheep (identified as D4, D5, D6 and D7) were orally challenged with PG127 classical scrapie isolate (total dose 10^6.7^ ID_50_ IC in tg338 mice). Scrapie incubation period in sheep were respectively 226 days, 238 days, 228 days and 242 days post inoculation (dpi). Aliquot of the same fresh and PFA 2% fixed WBC than those IV administrated sheep (Table 5) were homogenised in 5% glucose before intracerebral inoculation in tg338 mice (n = 6 per sample, 20 µL per mice). Each mouse received a quantity of WBC that is equivalent to 2.5 mL of starting whole blood. Mice were observed till occurrence of clinical signs compatible with a transmissible spongiform encephalopathy and considered positive when abnormal PrP deposition was detected in brain. Incubation periods (days post inoculation: dpi) in mice are presented as mean +/−SD except for those dilutions with which less than 50% of mice were found positive. In that case individual incubation period are reported (†). Infectious titers were estimated by the Spearman-Karber method [19]. Infectious titer is expressed as number of ID_50_ per mL of whole blood. For each samples, the most likely value and, in parentheses, the lower and upper value of the 95% confidence interval are reported.

Whole blood and fresh WBC produced clinical scrapie in all recipients. The incubation periods of recipient sheep receiving whole blood or WBC were similar suggesting comparable virulence of both products (Table 4). In contrast, the intravenous administration of crude plasma resulted in infection of only three of the four recipient sheep. The incubation periods of the plasma infections were substantially longer than in sheep who received whole blood. None of the sheep that received plasma that had been prepared from 20 mL of whole blood were infected. Donor (D4), whose plasma did not transmit the disease (even when prepared from 200 ml of whole blood), was also part of the IV whole blood titration (Table 2) where 0.2 mL of its whole blood was sufficient to infect the recipient. From this it can be concluded that, in this individual, plasma was at least 10,000 fold less efficient than whole blood for transmitting the disease by the intravenous route.

Whereas all recipients that received fresh WBC succumbed to clinical disease, the IV administration of PFA fixed WBCs resulted in TSE infection of only two of four recipient sheep (Table 5) and in incubation periods that exceeded those observed for plasma recipients. This outcome might suggest that PFA fixation inactivated WBC associated infectivity. However, when inoculated IC into tg338 mice, the same fresh and PFA fixed WBC homogenates produced 100% attack rates with similar incubation periods for both groups (Table 6). The end point titration by IC inoculation in tg338 of PFA fixed and fresh WBC homogenates from two of these donors (D4 and D7) further confirmed that the fixation procedure we applied did not substantially affected the WBC's infectious titer (Table 6).

## Discussion

Using TSE infection in sheep as a model, we have shown that the intravenous administration of a few hundred microliters of blood is sufficient to infect a transfusion recipient. This high efficiency of transmission by blood transfusion contrasts with the very low infectious titer measured by IC inoculation of blood into transgenic mice expressing ovine PrP.

This discrepancy shows that the infectious titer as determined by IC inoculation of rodents does not accurately predict the ability of blood and blood-derived products to transmit the disease when administered by the intravenous route. Importantly, this finding indicates that assessments of transfusion transmission risks from v-CJD that are based on IC measurements in rodents may seriously underestimate the risk.

Our results indicate that freshly prepared WBCs have a comparable capacity to whole blood to infect transfusion recipients. However WBC fixation with PFA strongly inhibited their ability to transmit the disease intravenously. Tissue fixation with aldehydes is classically described to reduce by several log10 the TSE infectivity titer (as measured by IC inoculation in rodents) [24], [25]. However this activity is dependent on the fixation procedure and in particular on its duration. For instance, while the fixation of a 263 K scrapie 10% brain homogenate with 3.7% formaldehyde during 4 hours (at room temperature) resulted in a 1.58 log10 drop in the infectivity, a 30 min fixation had no significant impact on the titer [26]. Our bioassay experiments in tg338 mice, confirmed that the fixation procedure we applied (2% PFA during 10 minutes on ice) preserved TSE infectivity. Beside this, PFA fixation stabilizes cellular morphology through cross linking of the cell surface proteins which also makes it impossible for cells to change their cell membrane molecules and impairs their capacity to develop dynamic cellular interactions [27], [28]. We therefore think that the ability of blood (or blood-derived products) to transmit the disease by the transfusion route crucially depends on the ability of blood cells to interact with the host.

This view is consistent with results obtained in *in vitro* prion infection models in which cell-mediated infection was reported to be more efficient than infection with cell homogenates or PFA fixed cells [29], [30]. It also concurs with the previously reported ability of living splenic cells prepared from scrapie infected C57Bl6 mice to transmit scrapie in *RAG-1^0/0^* mice following IV administration whereas no transmission could be observed following the IV administration of splenic cell homogenates to the same mouse model [31]. Whether the high capability of viable WBC to infect blood recipients relies on the capacity of certain cellular populations to deliver TSE agent to specific target site(s) will require further investigation.

In the PG127 scrapie sheep model, plasma was less able than whole blood and fresh WBCs to transmit the disease by the transfusion route. This observation is consistent with data obtained from similar transfusion experiments carried out in BSE infected sheep and in Chronic Wasting Disease infected cervids [32], [33]. The low transmissibility of the disease by the plasma in these animal models contrasts with the results obtained in rodents where plasma contains 40 to 60% of the infectivity in whole blood [7], [8], [34]. Together, with the infectivity measurements that we performed, these data support the contention that the partitioning of TSE agents in blood components differs between sheep and rodents models. However, the differences of infectivity load between plasma and WBC that we observed are unlikely to explain on their own the limited efficacy of plasma to transmit the disease (by comparison to whole blood and WBC). This statement is strongly supported by the WBC fixation experiment that we performed in which the PFA treatment did not alter WBCs infectious titer but reduced their capacity to transmit the disease to recipients at level which is comparable to plasma.

In this study, labile blood products containing viable WBC presented the greatest risk of transmitting Prion disease. This finding strongly supports the continuation of universal leuco-reduction as currently applied in the EU countries and Canada to reduce, amongst other potential adverse effects, the risk of v-CJD transmission [34]. Additional experiments will be necessary to determine the minimal number of WBC (leukocytes and/or platelets) that is sufficient to transmit the disease and to identify the WBC cell population(s) responsible for virulence.

Finally, our results also raise some concerns about the use of the ‘spiking’ models for investigation of blood-borne TSE transmission risk [35], [36]. Whereas this approach is very convenient to measure the TSE infectivity reduction by certain process, it is probably of limited relevance for assessing the efficacy of devices intended to mitigate the risk of Prion disease transmission by blood and blood derived products.

## References

[1] Bruce ME, Will RG, Ironside JW, McConnell I, Drummond D (1997). Transmissions to mice indicate that ‘new variant’ CJD is caused by the BSE agent.. Nature.

[2] Collinge J, Sidle KC, Meads J, Ironside J, Hill AF (1996). Molecular analysis of prion strain variation and the aetiology of ‘new variant’ CJD.. Nature.

[3] Llewelyn CA, Hewitt PE, Knight RS, Amar K, Cousens S (2004). Possible transmission of variant Creutzfeldt-Jakob disease by blood transfusion.. Lancet.

[4] Lefrere JJ, Hewitt P (2009). From mad cows to sensible blood transfusion: the risk of prion transmission by labile blood components in the United Kingdom and in France.. Transfusion.

[5] Gregori L, McCombie N, Palmer D, Birch P, Sowemimo-Coker SO (2004). Effectiveness of leucoreduction for removal of infectivity of transmissible spongiform encephalopathies from blood.. Lancet.

[6] Brown P, Rohwer RG, Dunstan BC, MacAuley C, Gajdusek DC (1998). The distribution of infectivity in blood components and plasma derivatives in experimental models of transmissible spongiform encephalopathy.. Transfusion.

[7] Brown P, Cervenakova L, McShane LM, Barber P, Rubenstein R (1999). Further studies of blood infectivity in an experimental model of transmissible spongiform encephalopathy, with an explanation of why blood components do not transmit Creutzfeldt-Jakob disease in humans.. Transfusion.

[8] Cervenakova L, Yakovleva O, McKenzie C, Kolchinsky S, McShane L (2003). Similar levels of infectivity in the blood of mice infected with human-derived vCJD and GSS strains of transmissible spongiform encephalopathy.. Transfusion.

[9] Holada K, Vostal JG, Theisen PW, MacAuley C, Gregori L (2002). Scrapie infectivity in hamster blood is not associated with platelets.. J Virol.

[10] Gregori L, Gurgel PV, Lathrop JT, Edwardson P, Lambert BC (2006). Reduction in infectivity of endogenous transmissible spongiform encephalopathies present in blood by adsorption to selective affinity resins.. Lancet.

[11] Houston F, Foster JD, Chong A, Hunter N, Bostock CJ (2000). Transmission of BSE by blood transfusion in sheep.. Lancet.

[12] Hunter N, Foster J, Chong A, McCutcheon S, Parnham D (2002). Transmission of prion diseases by blood transfusion.. J Gen Virol.

[13] Ironside JW, Bishop MT, Connolly K, Hegazy D, Lowrie S (2006). Variant Creutzfeldt-Jakob disease: prion protein genotype analysis of positive appendix tissue samples from a retrospective prevalence study.. BMJ.

[14] Ironside JW, Hilton DA, Ghani A, Johnston NJ, Conyers L (2000). Retrospective study of prion-protein accumulation in tonsil and appendix tissues.. Lancet.

[15] Simmons HA, Simmons MM, Spencer YI, Chaplin MJ, Povey G (2009). Atypical scrapie in sheep from a UK research flock which is free from classical scrapie.. BMC Vet Res.

[16] Andreoletti O, Orge L, Benestad SL, Beringue V, Litaise C (2011). Atypical/Nor98 scrapie infectivity in sheep peripheral tissues.. PLoS Pathog.

[17] Arsac JN, Andreoletti O, Bilheude JM, Lacroux C, Benestad SL (2007). Similar biochemical signatures and prion protein genotypes in atypical scrapie and Nor98 cases, France and Norway.. Emerg Infect Dis.

[18] Le Dur A, Beringue V, Andreoletti O, Reine F, Lai TL (2005). A newly identified type of scrapie agent can naturally infect sheep with resistant PrP genotypes.. Proc Natl Acad Sci U S A.

[19] Markus RA, Frank J, Groshen S, Azen SP (1995). An alternative approach to the optimal design of an LD50 bioassay.. Stat Med.

[20] Fisher RA (1936). Uncertain Inference.. P Am Acad Arts Sci.

[21] Gregori L, Lambert BC, Gurgel PV, Gheorghiu L, Edwardson P (2006). Reduction of transmissible spongiform encephalopathy infectivity from human red blood cells with prion protein affinity ligands.. Transfusion.

[22] Lacroux C, Corbiere F, Tabouret G, Lugan S, Costes P (2007). Dynamics and genetics of PrPSc placental accumulation in sheep.. J Gen Virol.

[23] Brown P, Cervenakova L, McShane LM, Barber P, Rubenstein R (1999). Further studies of blood infectivity in an experimental model of transmissible spongiform encephalopathy, with an explanation of why blood components do not transmit Creutzfeldt-Jakob disease in humans.. Transfusion.

[24] Brown P, Liberski PP, Wolff A, Gajdusek DC (1990). Resistance of scrapie infectivity to steam autoclaving after formaldehyde fixation and limited survival after ashing at 360 degrees C: practical and theoretical implications.. J Infect Dis.

[25] Wadsworth JD, Dalmau-Mena I, Joiner S, Linehan JM, O'Malley C (2010). Effect of fixation on brain and lymphoreticular vCJD prions and bioassay of key positive specimens from a retrospective vCJD prevalence study.. J Pathol.

[26] Brown P, Rohwer RG, Green EM, Gajdusek DC (1982). Effect of chemicals, heat, and histopathologic processing on high-infectivity hamster-adapted scrapie virus.. J Infect Dis.

[27] Kawakami K, Kakimoto K, Shinbori T, Onoue K (1989). Signal delivery by physical interaction and soluble factors from accessory cells in the induction of receptor-mediated T-cell proliferation. Synergistic effect of BSF-2/IL-6 and IL-1.. Immunology.

[28] Peguet-Navarro J, Dalbiez-Gauthier C, Schmitt D (1992). Accessory function of human Langerhans cells in the primary allogeneic T-cell response.. J Invest Dermatol.

[29] Kanu N, Imokawa Y, Drechsel DN, Williamson RA, Birkett CR (2002). Transfer of scrapie prion infectivity by cell contact in culture.. Curr Biol.

[30] Paquet S, Langevin C, Chapuis J, Jackson GS, Laude H (2007). Efficient dissemination of prions through preferential transmission to nearby cells.. J Gen Virol.

[31] Aucouturier P, Geissmann F, Damotte D, Saborio GP, Meeker HC (2001). Infected splenic dendritic cells are sufficient for prion transmission to the CNS in mouse scrapie.. J Clin Invest.

[32] McCutcheon S, Alejo Blanco AR, Houston EF, de Wolf C, Tan BC (2011). All Clinically-Relevant Blood Components Transmit Prion Disease following a Single Blood Transfusion: A Sheep Model of vCJD.. PLoS One.

[33] Mathiason CK, Hayes-Klug J, Hays SA, Powers J, Osborn DA (2010). B cells and platelets harbor prion infectivity in the blood of deer infected with chronic wasting disease.. J Virol.

[34] Vamvakas EC (2011). Universal white blood cell reduction in Europe: has transmission of variant Creutzfeldt-Jakob disease been prevented?. Transfus Med Rev.

[35] Morales R, Buytaert-Hoefen KA, Gonzalez-Romero D, Castilla J, Hansen ET (2008). Reduction of prion infectivity in packed red blood cells.. Biochem Biophys Res Commun.

[36] Cardone F, Simoneau S, Arzel A, Puopolo M, Berardi VA (2012). Comparison of nanofiltration efficacy in reducing infectivity of centrifuged versus ultracentrifuged 263 K scrapie-infected brain homogenates in “spiked” albumin solutions.. Transfusion.

